# Homœpathia

**Published:** 1837

**Authors:** 


### Art. 5.—Homœopathia.

*Homœopathic Medicine;* Illustrating its superiority over the other Medical Doctrines, with an account of the Regimen to be followed during the treatment of Diseases. By M. Croserio, M. D., President of the Homœopathic Society of Paris, &c. &c. Translated from the French, with Notes, by C. Neidhard, M. D.—12mo. pp. 89. Philadelphia: 1837.

This is, in many respects, a most extraordinary and singular little book; extraordinary for its bold and unsupported assertions, singular for its enthusiastic praise of a system of medicine which bears upon its very front the impress of folly and destruction. It has been said by an able divine, that there is no doctrine in religion, however absurd, that has not had its admirers and proselytes, and the same, we are sure, is eminently true of medicine and its kindred sciences. Centuries elapsed before physicians were able to break the fetters which had been bound around them by Galen; years passed before they perceived the fallacies of the bewitching theories and hypotheses of Boerhaave, of Darwin, of Brown, and of Cullen; and many a sun will sink into the dark shades of night before they will be able to free themselves from the authority of a Broussais, a Hahnemann, and a Rush.

To the vulgar there is always something peculiarly fascinating in dogmatism, and when a man asserts upon every page of his book, "*I speak from experience*," he is as sure of his game as Colonel Crockett was when he grinned at a squirrel. Authority with the unthinking part of mankind is everything; it is a sort of bastinado, from which there is no appeal. "Ich rede aus erfahrung," why the phrase is absolutely irresistible. Every reader of Hahnemann is convinced, and whilst staring with admiration at the sublime truths of the "new doctrine," he gulps down at one effort the whole materia medica of the German mystic.

We have said that Dr. Croserio's book is extraordinary for its impudence and bold assertions. To substantiate this remark, it is only necessary to quote his own language. Thus, at the close of the work, in summing up the reciprocal advantages of homœopathia and the old medical doctrines, as he is pleased to style them, the author makes the following statement, which we shall present to our readers in full.

| | |
|---|---|
| "1. The homœopathic physician investigates most accurately every circumstance calculated to throw light on the nature of the disease, in order to seize its particular characteristics, and he does not permit his imagination to mislead him by suppositions. | "1. The allœopathist is satisfied with the investigation of the general character and species of the disease and of its nature according to the prevailing theory. |
| "2. He always employs agents which have been previously tried, and with whose effects on the healthy body he is perfectly familiar. | "2. He only employs remedies with which chance has furnished him, or which have been tried merely in diseases of the same name, or according to virtues which his imagination suggests to him. |

| | |
|---|---|
| "3. He always exhibits medicines without any admixture, which might disturb their action. | "3. He exhibits several substances at the same time, which will reciprocally destroy one another, or produce an uncertain and irregular effect. |
| "4. He employs medicines in the smallest doses, and at prolonged intervals. | "4. He uses them in very strong doses and at short intervals. |
| "5. He does not spill the blood and animal fluids; nor does he torment the patient with blisters, moxa, &c. | "5. He spills the blood and other animal fluids, and torments the sick by painful irritants which enfeeble and retard the healing powers of nature and exhaust it. |
| "6. He prescribes a simple regimen conformable to the laws of nature, endeavoring to preserve the strength of the patient, so as to make no period of convalescence necessary, or to render it very short. | "6. He prescribes total abstinence from food, or a choice of aliments little appropriate to the function of the stomach, enfeebling the patient and rendering the cure very slow and difficult, and the convalescence endless. |

In winding up this comparison, which reminds one of the fly and the ox in the fable, our argumentative Croserio adds, by way of capping the climax, the following delectable morceau: "The homœopathist attains therefore, as nearly as possible, all the conditions prescribed by Celsus for a good cure, *tuto*, *cito*, *et jucunde*;" in plain stage-coach English, with safety, dispatch, and gentleness. This, indeed, is going at a most rapid rate, and if the allœopathist escapes, he may thank his stars rather than the complacent disciples of Samuel Hahnemann.

In speaking of bleeding, of which, in common with all homœopathists, he has evidently a great horror, Dr. Croserio whines and laments in the true style of a modern philanthropist. It has been said by some antiquarian, that Adam was above a hundred and thirty feet high, and that our author, and for aught we know to the contrary, the whole tribe of homœopathists, are firm believers in this doctrine, fully appears from the subjoined quotation. "Is it to be wondered at, then," says he, "that mankind degenerate, that the human size becomes smaller and smaller, and the limbs less strong, that the powers of the mind become weakened, when we see a fluid, on which depend the nutrition, growth, and vigor of the body, so rashly spilled." This, indeed, is a serious matter, and calls loudly for reform. To abstract the vital fluid we have long deemed an outrage upon society, and unless we wish all our children to become Lilliputians, it should no longer be tolerated by the people. Bleeding weakens the body, and stints the growth; just as the retention of the blood invigorates, expands, and elongates it. In truth, this is a thought worthy of the gods, and for which the Great Frederick of Prussia would have given half his dominions. Dear mothers, only retain your blood, and your offspring will soon rival their great progenitor, Adam. By the way,—and we put the query in all soberness,—is not bleeding the cause of man's depravity and wickedness? Would not physicians be less liable to quarrel if they no longer lost blood by the lancet?

"It was time," says Dr. Croserio, "in order to prevent the complete degradation of the human race, that a more rational system of medicine should succeed one productive of such pernicious practice. To prevent our total destruction, God, in his infinite goodness, has raised up the founder of homœopathia, to heal diseases with remedies more in conformity with the intentions of nature, and to re-establish health without destroying the source of life." "This," adds the translator, "is undoubtedly very true. Several homœopathic physicians have already observed in their practice, that all children, born after the parents had been subjected to homœopathic treatment, were generally much stronger, healthier, and less liable to disease, than those born whilst the parents were treated in the old way. We may therefore justly entertain the hope, that with the extension of homœopathia, a stronger, and certainly also, a more noble generation will rise up in future time."

Such are a few specimens of the arrogance and self-conceit of this Frenchified homœopathist; the espouser of a foolish system, which promises to cure all diseases, to prolong the term of human existence, and to restore mankind to their primitive altitude and mental powers. For such purposes has God sent Hahnemann! Was there ever more absurd bombast, more outrageous flummery, more extravagant folly? Hohenloeism is a pigmy to it; Thompsonianism a mere puff.

Hahnemann, nearly ninety years of age, now resides in Paris, where he has erected the standard of homœopathia, and patiently waits the fulfilment of the prophecy which he uttered many years ago, *that from France would issue that general impulse which would render his discovery popular in all parts of the world!* Long may the monomaniac live to enjoy the fruits of his labors, and may France, rather than the United States, become the theatre of his disciples. S. D. G.

